# The mitogenome of common snipe, *Gallinago gallinago gallinago* Linnaeus, 1758 and evolutionary implications for the family Scolopacidae

**DOI:** 10.1080/23802359.2021.1972870

**Published:** 2021-09-09

**Authors:** Chao Yang, Xiang Hou, Bo-Ye Liu, Hui-Sheng Gong, Hao Yuan, Xue-Juan Li, Jie Tang, Yan Wang

**Affiliations:** a Shaanxi Institute of Zoology, Xi’an, China; b College of Life Sciences, Shaanxi Normal University, Xi’an, China; c School of Basic Medical Sciences, Xi’an Medical University, Xi’an, China

**Keywords:** Mitogenome, cytosine insertion, evolutionary implications, *Gallinago gallinago gallinago*

## Abstract

The mitochondrial genome (mitogenome) of *Gallinago gallinago gallinago* Linnaeus, 1758 was determined by the high-throughput data. The assembled mitogenome was 16,919 bp in length, with a 58.7% A + T content and GC skew of −0.3850. Among 13 PCGs, an unusual start codon (GTG) was identified for the *COX1* gene, and incomplete stop codons (T-) were found in the *COX3*, *ND2* and *ND4* genes. The function of a cytosine insertion at site 174 in the *ND3* gene and its phylogenetic significance are worthy of further scrutiny. In the control region (*CR*), thirteen 15-bp simple sequence repeats were found in *G. g. gallinago*. Phylogenetic analysis indicated that *Gallinago* was clustered at the basal position of the *Scolopax* clade and that the monophyly of *Gallinago* was also recovered. The mitogenome data of *G. g. gallinago* provides useful resources for further studying the evolution of Scolopacidae.

The common snipe, *Gallinago gallinago*, is a medium-sized, ground-nesting shorebird that feeds by probing in mud or soil to locate invertebrates with its long bill. This species occurs on all continents except Antarctica and Australia, but populations have been declining in Europe because of the drainage of bogs and marshy grassland (Cramp and Simmons 1983). Most species of birds separate molting from other energy-demanding activities, such as migration or reproduction. *G. gallinago* is an exception, as during the first autumn migration, many young snipe initiate their post-juvenile molt, which includes the replacement of body feathers, lesser and median wing coverts, tertials, and rectrices (Podlaszczuk et al. 2017). *G. gallinago* is a grassland waterbird characteristic of agricultural meadows and a member of one of the most threatened bird guilds (Regos et al. 2020). As an indicator species of the ecological environment, research on *G. gallinago* has mostly focused on macroscopic ecology (Green 1988; Henderson et al. 2002; Włodarczyk et al. 2018). Limited molecular data hamper phylogenetic and evolutionary studies in *G. gallinago*. Here, the complete mitogenome of *G. g. gallinago* has been reported with general features. These results are used to explore the potential for improving breeding success by habitat management.

*G. g. gallinago* in this study was a female adult that died naturally during the breeding season from Lantian County, Xi’an (34°20′7ʺN, 109°22′54ʺE). The specimen (voucher number: SWSZ01) was collected and identified by C. Yang, and deposited in the animal specimen museum of Shaanxi Institute of Zoology, Xi’an, Shaanxi Province, China (contacts: Chao Yang, chaoy819@xab.ac.cn).

DNA was extracted by Qiagen columns (DNeasy^®^ Blood & Tissue kit; Qiagen, Hilden, Germany) and prepared with a paired-end (2 × 150 bp) library strategy followed by next-generation sequencing (NGS) on the Illumina HiSeq Xten platform (Illumina, San Diego, CA). A total of 9,219,657 paired-end raw reads were produced. After removing regions with a Phred score of <10, 9,111,941 clean reads were obtained after quality and ambiguity controls. The clean data were assembled using MITOBim v1.9 (Hahn et al. 2013) with the mitogenome of *G. stenura* (GenBank accession no. KY056596) as a reference. Assembly of the clean reads and gene annotation were performed by Geneious 10.1.3 (Kearse et al. 2012) and tRNAscan-SE 2.0 (Lowe and Chan 2016), with a total of 210,657 mitochondrial reads mapped to the reference mitogenome, giving an average coverage of 1820.1×.

The assembled *G. g. gallinago* mitogenome (GenBank accession no. MZ157405) was a 16,919 bp long circular DNA with overall nucleotide frequencies of A = 32.5%, T = 26.2%, C = 28.6%, and G = 12.7% and an A + T content of 58.7%. The GC skew was −0.3850, which showed a remarkable C skew and was similar to the mitogenomes of other Charadriiformes species (Yu et al. 2014; Yang et al. 2016). With the exception of *COX1*, which started with GTG, all PCGs had typical ATN start codons, and all PCGs ended with a complete triplet codon (TAA, AGG, AGA, or TAG), except for *COX3*, *ND2*, and *ND4*, which ended with an incomplete T. All transfer RNA (tRNA) genes had typical cloverleaf secondary structures, with the exception of *tRNA-Ser* (AGY), in which the dihydrouridine arm formed a simple loop. The length of *12S ribosomal RNA* (rRNA) was 972 bp, and that of *16S rRNA* was 1598 bp, both were located between *tRNA-Phe* and *tRNA-Leu* (UUR) and separated by *tRNA-Val*. The *CR* was 1364 bp long and was located between *tRNA-Glu* and *tRNA-Phe*.

The newly sequenced species with 99.3% sequence similarity to *G. gallinago* (GB: MW865755), and the nucleotide differences between of them occurred mainly in the CR: length of poly-C block in domain I and number of repeat units in domain III (Figure 1(A)). In the *CR*, 13 (positions: 16,691–16,885) and 8 (positions: 16,680–16,799) simple sequence repeats of 5′-AAACAAACAATCAAC-3′ existed in *G. g. gallinago* (GB: MZ157405) and *G. gallinago* (GB: MW865755), respectively (software: tandem repeats finder (TRF) v4.09; Benson 1999). This is the main reason for the different lengths of the two sequences. More molecular sequence data generated may be applied to elucidate the population genetics of this bird species.

**Figure 1.**
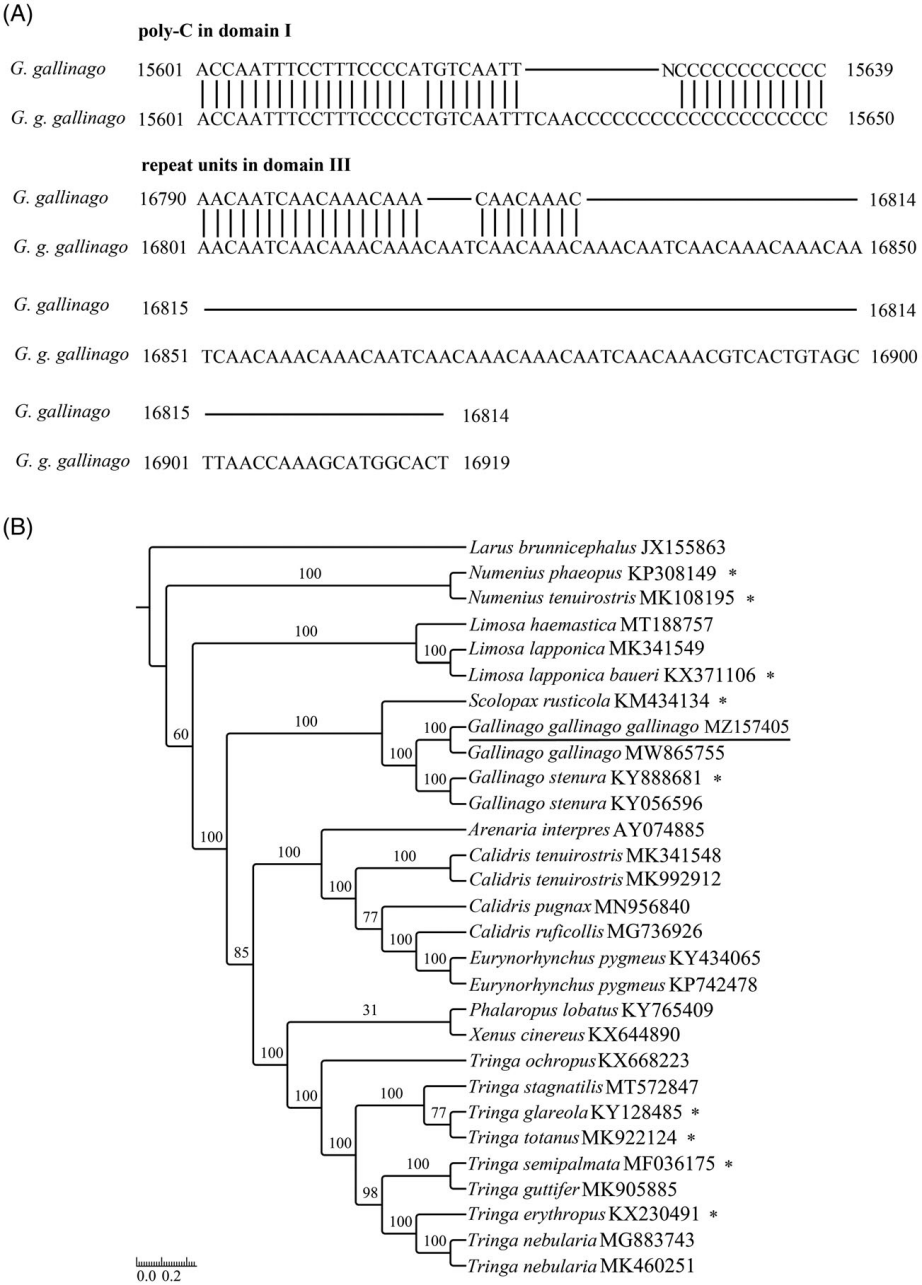
(A) Main nucleotide trait differences between *G. gallinago* (GenBank accession no. MW865755, MZ157405). (B) Maximum-likelihood tree obtained using RAxML v7.0.3 with 1000 nonparametric bootstrap replicates. GenBank accession numbers are indicated following species names. Numbers on the nodes are bootstrap values. ‘_*_’ represents no cytosine insertion.

The length of the *ND3* gene was 351 bp, which was similar to most of the known Scolopacidae mitogenomes in GenBank. One cytosine insertion at site 174 was revealed in the *ND3* gene (nucleotide position: 9703). Classic inference suggests that this extra nucleotide may be removed by RNA editing during translation, and the function of the *ND3* gene may be recovered, effectively avoiding the premature stoppage of transcription due to a frameshift mutation (Mindell et al. 1998). The latest hypotheses expounded that relaxed selection pressure may have allowed frameshift insertions to be tolerated for hundreds of millions of years, possibly a result of the rapid adaptive radiation of birds due to programmed translational frameshifting (Rosengarten et al. 2008; Russell and Beckenbach 2008; Haen et al. 2014). Species without cytosine insertion in the *ND3* gene of Scolopacidae have been labeled with ‘_*_’ (Figure 1(B)). At the order level, species with cytosine insertion in the *ND3* gene (Struthioniformes) were clustered into one branch and species without cytosine insertion in the *ND3* gene clustered into another branch (Passeriformes). Species with cytosine insertion in the *ND3* gene were so antiquated that they were divided out earlier. Species with/without cytosine insertion in the *ND3* gene (Tinamiformes, Falconiformes, Charadriiformes, Sphenisciformes, Ciconiiformes, Galliformes, Anseriformes, etc.) clustered at the neutral position of the avian mitogenomic tree (Slack et al. 2007; Tamashiro et al. 2019). But at the family level, it seems that the clustering of related species are not hindered by cytosine insertion in the *ND3* gene (Figure 1(B)). The mechanism of the extra 'C' in the *ND3* gene and its phylogenetic significance on avian adaptive radiation need to be studied further.

To validate the phylogenetic position of *G. g. gallinago*, maximum-likelihood (ML) methods were employed to construct phylogenetic tree using RAxML v7.0.3 (Stamatakis 2006) based on 13 PCGs of 29 mitogenomes. The 13 PCGs sequences were aligned using Clustal v2.1 after manually removed the stop codons, and then concatenated into a combined dataset using SequenceMatrix v1.7.8. The best partitioning scheme and optimal model (models GTR + I + G and GTR + G) were analyzed in Partitionfinder v1.1.1 (Lanfear et al. 2012), and the robustness of the phylogenetic result was tested through bootstrap analysis with 1000 replicates (Yang et al. 2017). *Larus brunnicephalus* (GenBank accession no. JX155863) was selected as an outgroup. The topological structure showed that the monophyly of *Gallinago* was recovered, with the phylogeny ((*G. gallinago* (GB: MW865755), *G. g. gallinago* (GB: MZ157405)) (*G. stenura* (GB: KY888681), *G. stenura* (GB: KY056596))) (Figure 1(B)) (Gibson and Baker 2012). We also confirmed that *Gallinago* was clustered at the basal position of the *Scolopax* clade (Figure 1(B)), which was consistent with the results of previous studies (Hu et al. 2017). This mitogenome would provide an important resource for further exploring the taxonomic status of Scolopacidae species.

## Data Availability

The genome sequence data that support the findings of this study are openly available in NCBI GenBank (https://www.ncbi.nlm.nih.gov/) under accession no. MZ157405. The associated BioProject, BioSample, and SRA numbers are PRJNA727369, SAMN19016979, and SRR14424283, respectively.
